# Supplementary material for: Pathogenesis and defense mechanism while Beauveria bassiana JEF-410 infects poultry red mite, Dermanyssus gallinae
Source: PLoS One. 2023 Feb 17;18(2):e0280410. doi: 10.1371/journal.pone.0280410 (PMC9937463; doi:10.1371/journal.pone.0280410)
Supplement: S1 Fig — (PPTX) [file pone.0280410.s001.pptx]

## Slide 1
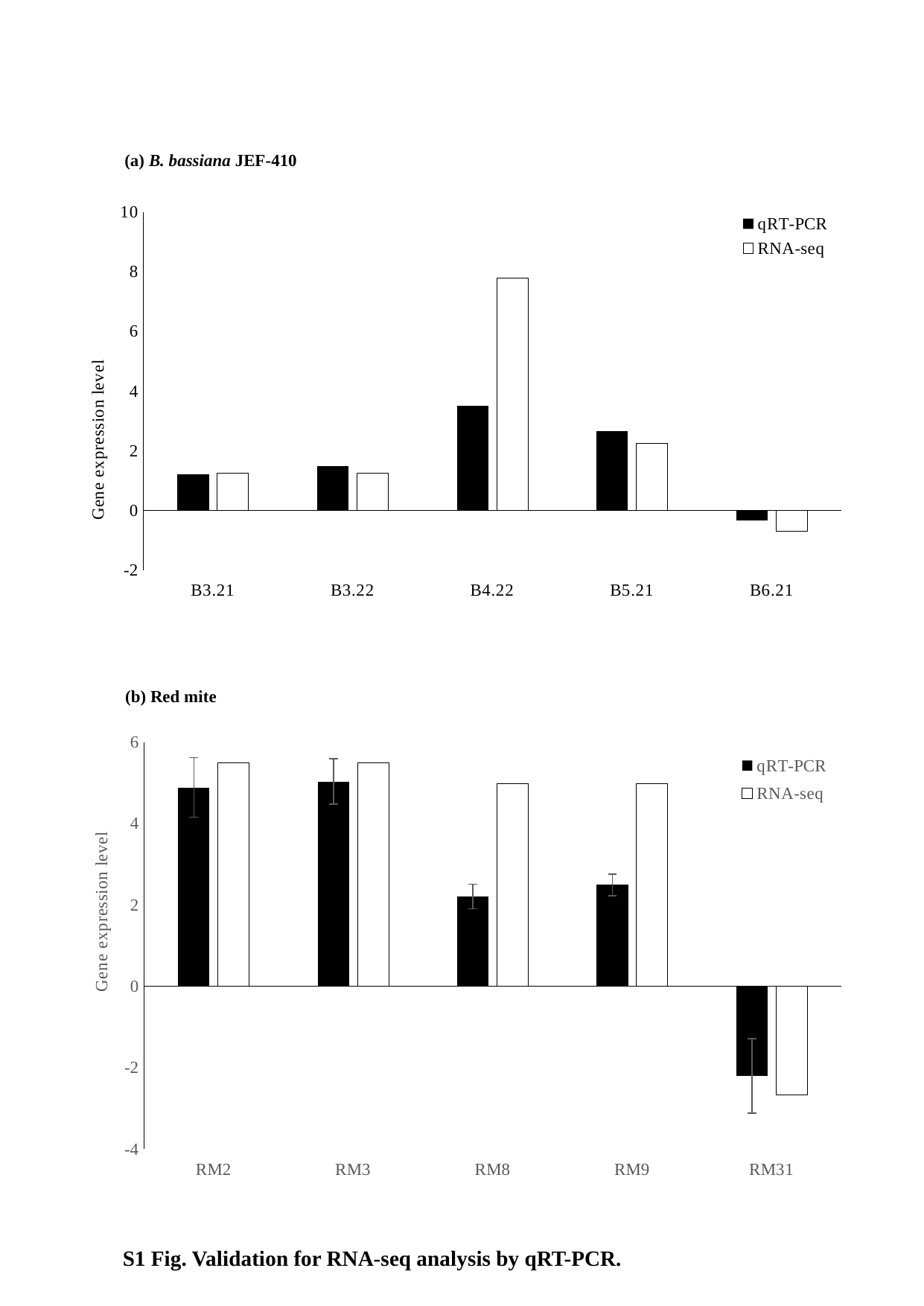

(a) B. bassiana JEF-410
### Chart
| Category | qRT-PCR | RNA-seq |
|---|---|---|
| B3.21 | 1.2069806220273378 | 1.24379800956616 |
| B3.22 | 1.4838143463087548 | 1.24379800956616 |
| B4.22 | 3.516209385028457 | 7.78150437563009 |
| B5.21 | 2.6553324244869643 | 2.24566918237071 |
| B6.21 | -0.3349431535619978 | -0.706156813843571 |(b) Red mite
### Chart
| Category | qRT-PCR | RNA-seq |
|---|---|---|
| RM2 | 4.88890641698747 | 5.49395 |
| RM3 | 5.034002954694169 | 5.49395 |
| RM8 | 2.207240824201272 | 4.98632 |
| RM9 | 2.4932710268080043 | 4.98632 |
| RM31 | -2.2009831191049223 | -2.6757 |S1 Fig. Validation for RNA-seq analysis by qRT-PCR.
