# Supplementary material for: Pathogenesis and defense mechanism while Beauveria bassiana JEF-410 infects poultry red mite, Dermanyssus gallinae
Source: PLoS One. 2023 Feb 17;18(2):e0280410. doi: 10.1371/journal.pone.0280410 (PMC9937463; doi:10.1371/journal.pone.0280410)
Supplement: S1 Table — (PPTX) [file pone.0280410.s002.pptx]

## Slide 1
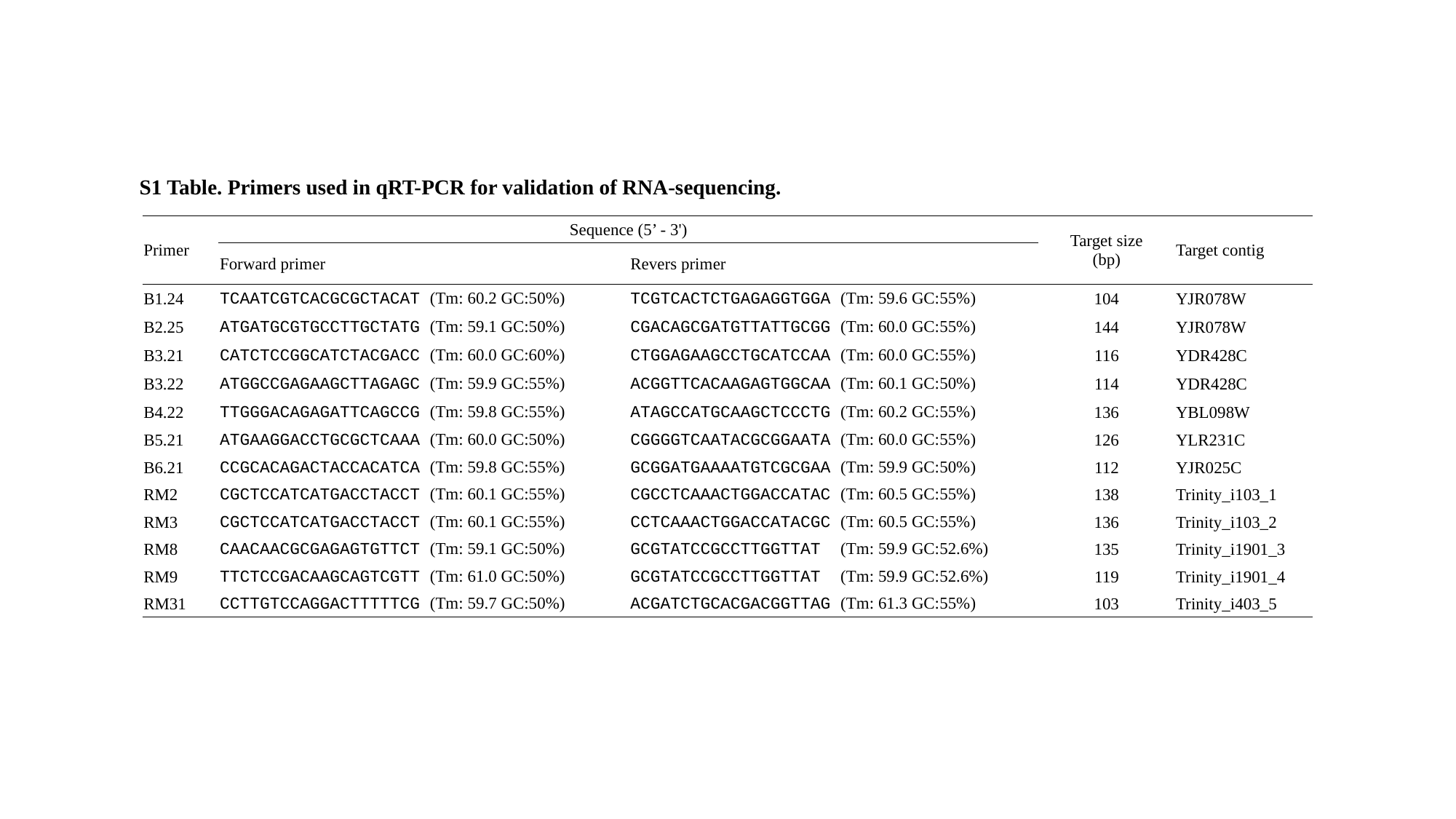

S1 Table. Primers used in qRT-PCR for validation of RNA-sequencing.
| Primer | Sequence (5’ - 3') | | Target size (bp) | Target contig |
| --- | --- | --- | --- | --- |
| | Forward primer | Revers primer | | |
| B1.24 | TCAATCGTCACGCGCTACAT (Tm: 60.2 GC:50%) | TCGTCACTCTGAGAGGTGGA (Tm: 59.6 GC:55%) | 104 | YJR078W |
| B2.25 | ATGATGCGTGCCTTGCTATG (Tm: 59.1 GC:50%) | CGACAGCGATGTTATTGCGG (Tm: 60.0 GC:55%) | 144 | YJR078W |
| B3.21 | CATCTCCGGCATCTACGACC (Tm: 60.0 GC:60%) | CTGGAGAAGCCTGCATCCAA (Tm: 60.0 GC:55%) | 116 | YDR428C |
| B3.22 | ATGGCCGAGAAGCTTAGAGC (Tm: 59.9 GC:55%) | ACGGTTCACAAGAGTGGCAA (Tm: 60.1 GC:50%) | 114 | YDR428C |
| B4.22 | TTGGGACAGAGATTCAGCCG (Tm: 59.8 GC:55%) | ATAGCCATGCAAGCTCCCTG (Tm: 60.2 GC:55%) | 136 | YBL098W |
| B5.21 | ATGAAGGACCTGCGCTCAAA (Tm: 60.0 GC:50%) | CGGGGTCAATACGCGGAATA (Tm: 60.0 GC:55%) | 126 | YLR231C |
| B6.21 | CCGCACAGACTACCACATCA (Tm: 59.8 GC:55%) | GCGGATGAAAATGTCGCGAA (Tm: 59.9 GC:50%) | 112 | YJR025C |
| RM2 | CGCTCCATCATGACCTACCT (Tm: 60.1 GC:55%) | CGCCTCAAACTGGACCATAC (Tm: 60.5 GC:55%) | 138 | Trinity\_i103\_1 |
| RM3 | CGCTCCATCATGACCTACCT (Tm: 60.1 GC:55%) | CCTCAAACTGGACCATACGC (Tm: 60.5 GC:55%) | 136 | Trinity\_i103\_2 |
| RM8 | CAACAACGCGAGAGTGTTCT (Tm: 59.1 GC:50%) | GCGTATCCGCCTTGGTTAT (Tm: 59.9 GC:52.6%) | 135 | Trinity\_i1901\_3 |
| RM9 | TTCTCCGACAAGCAGTCGTT (Tm: 61.0 GC:50%) | GCGTATCCGCCTTGGTTAT (Tm: 59.9 GC:52.6%) | 119 | Trinity\_i1901\_4 |
| RM31 | CCTTGTCCAGGACTTTTTCG (Tm: 59.7 GC:50%) | ACGATCTGCACGACGGTTAG (Tm: 61.3 GC:55%) | 103 | Trinity\_i403\_5 |
