# Supplementary material for: Pathogenesis and defense mechanism while Beauveria bassiana JEF-410 infects poultry red mite, Dermanyssus gallinae
Source: PLoS One. 2023 Feb 17;18(2):e0280410. doi: 10.1371/journal.pone.0280410 (PMC9937463; doi:10.1371/journal.pone.0280410)
Supplement: S2 Table — (PPTX) [file pone.0280410.s003.pptx]

## Slide 1
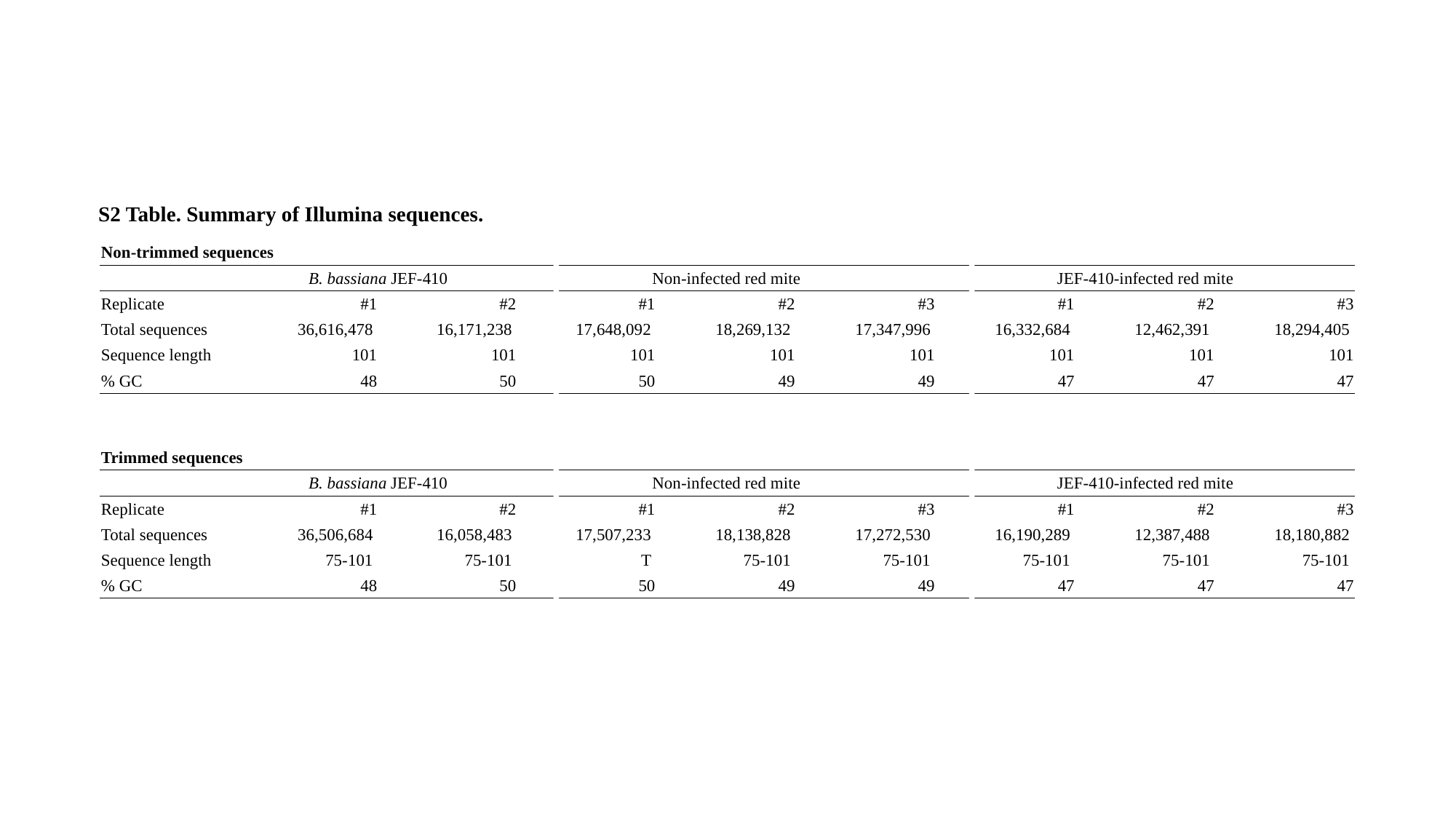

S2 Table. Summary of Illumina sequences.
| | | | | | | | | |
| --- | --- | --- | --- | --- | --- | --- | --- | --- |
| | | | | | | | | |
| Non-trimmed sequences | | | | | | | | |
| | B. bassiana JEF-410 | | Non-infected red mite | | | JEF-410-infected red mite | | |
| Replicate | #1 | #2 | #1 | #2 | #3 | #1 | #2 | #3 |
| Total sequences | 36,616,478 | 16,171,238 | 17,648,092 | 18,269,132 | 17,347,996 | 16,332,684 | 12,462,391 | 18,294,405 |
| Sequence length | 101 | 101 | 101 | 101 | 101 | 101 | 101 | 101 |
| % GC | 48 | 50 | 50 | 49 | 49 | 47 | 47 | 47 |
| | | | | | | | | |
| | | | | | | | | |
| Trimmed sequences | | | | | | | | |
| | B. bassiana JEF-410 | | Non-infected red mite | | | JEF-410-infected red mite | | |
| Replicate | #1 | #2 | #1 | #2 | #3 | #1 | #2 | #3 |
| Total sequences | 36,506,684 | 16,058,483 | 17,507,233 | 18,138,828 | 17,272,530 | 16,190,289 | 12,387,488 | 18,180,882 |
| Sequence length | 75-101 | 75-101 | T | 75-101 | 75-101 | 75-101 | 75-101 | 75-101 |
| % GC | 48 | 50 | 50 | 49 | 49 | 47 | 47 | 47 |
