# Supplementary material for: Pathogenesis and defense mechanism while Beauveria bassiana JEF-410 infects poultry red mite, Dermanyssus gallinae
Source: PLoS One. 2023 Feb 17;18(2):e0280410. doi: 10.1371/journal.pone.0280410 (PMC9937463; doi:10.1371/journal.pone.0280410)
Supplement: S3 Table — (PPTX) [file pone.0280410.s004.pptx]

## Slide 1
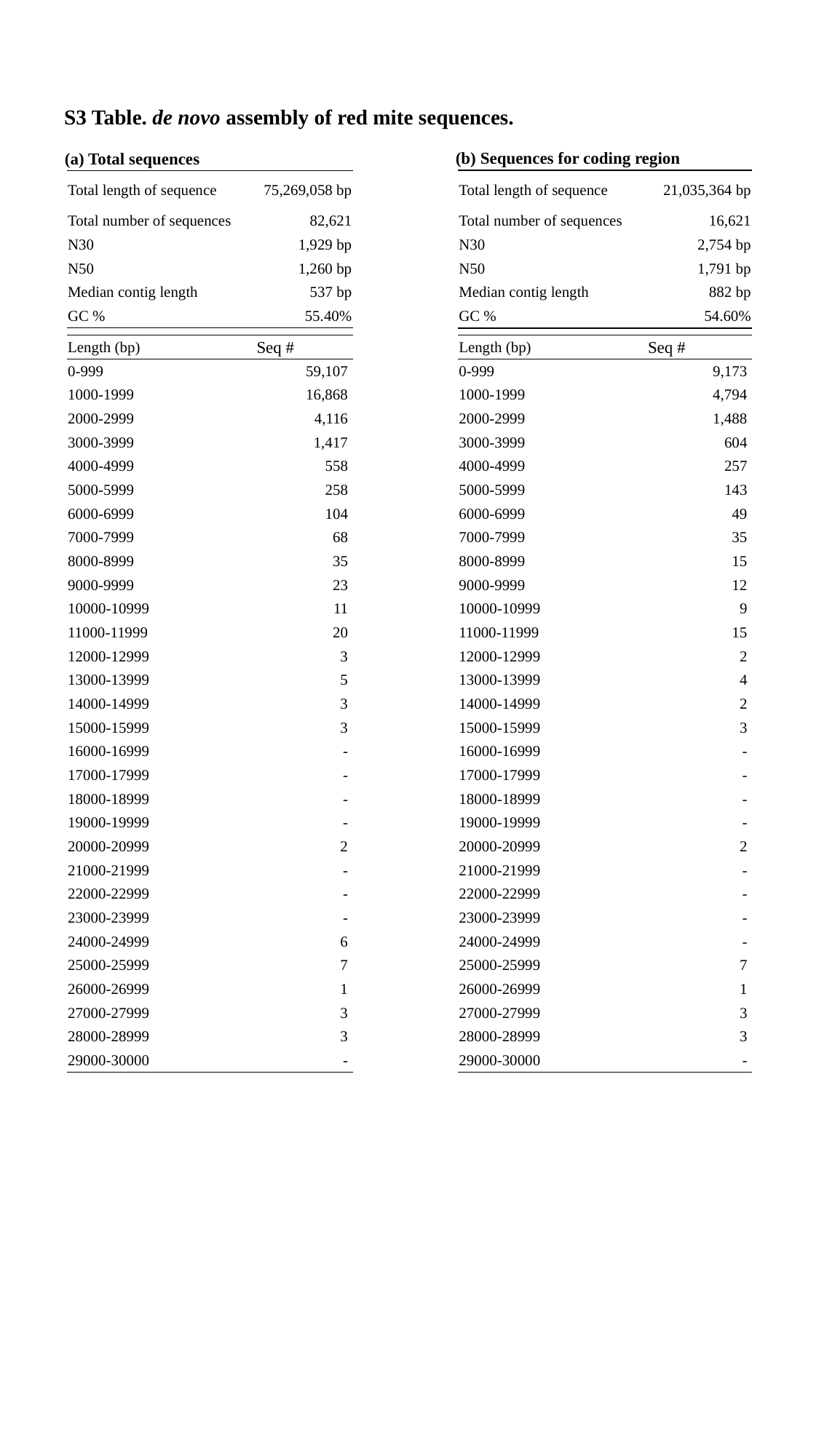

S3 Table. de novo assembly of red mite sequences.
(b) Sequences for coding region
(a) Total sequences
| Total length of sequence | 75,269,058 bp | | Total length of sequence | 21,035,364 bp |
| --- | --- | --- | --- | --- |
| Total number of sequences | 82,621 | | Total number of sequences | 16,621 |
| N30 | 1,929 bp | | N30 | 2,754 bp |
| N50 | 1,260 bp | | N50 | 1,791 bp |
| Median contig length | 537 bp | | Median contig length | 882 bp |
| GC % | 55.40% | | GC % | 54.60% |
| | | | | |
| Length (bp) | Seq # | | Length (bp) | Seq # |
| 0-999 | 59,107 | | 0-999 | 9,173 |
| 1000-1999 | 16,868 | | 1000-1999 | 4,794 |
| 2000-2999 | 4,116 | | 2000-2999 | 1,488 |
| 3000-3999 | 1,417 | | 3000-3999 | 604 |
| 4000-4999 | 558 | | 4000-4999 | 257 |
| 5000-5999 | 258 | | 5000-5999 | 143 |
| 6000-6999 | 104 | | 6000-6999 | 49 |
| 7000-7999 | 68 | | 7000-7999 | 35 |
| 8000-8999 | 35 | | 8000-8999 | 15 |
| 9000-9999 | 23 | | 9000-9999 | 12 |
| 10000-10999 | 11 | | 10000-10999 | 9 |
| 11000-11999 | 20 | | 11000-11999 | 15 |
| 12000-12999 | 3 | | 12000-12999 | 2 |
| 13000-13999 | 5 | | 13000-13999 | 4 |
| 14000-14999 | 3 | | 14000-14999 | 2 |
| 15000-15999 | 3 | | 15000-15999 | 3 |
| 16000-16999 | - | | 16000-16999 | - |
| 17000-17999 | - | | 17000-17999 | - |
| 18000-18999 | - | | 18000-18999 | - |
| 19000-19999 | - | | 19000-19999 | - |
| 20000-20999 | 2 | | 20000-20999 | 2 |
| 21000-21999 | - | | 21000-21999 | - |
| 22000-22999 | - | | 22000-22999 | - |
| 23000-23999 | - | | 23000-23999 | - |
| 24000-24999 | 6 | | 24000-24999 | - |
| 25000-25999 | 7 | | 25000-25999 | 7 |
| 26000-26999 | 1 | | 26000-26999 | 1 |
| 27000-27999 | 3 | | 27000-27999 | 3 |
| 28000-28999 | 3 | | 28000-28999 | 3 |
| 29000-30000 | - | | 29000-30000 | - |
