# Supplementary material for: Pathogenesis and defense mechanism while Beauveria bassiana JEF-410 infects poultry red mite, Dermanyssus gallinae
Source: PLoS One. 2023 Feb 17;18(2):e0280410. doi: 10.1371/journal.pone.0280410 (PMC9937463; doi:10.1371/journal.pone.0280410)
Supplement: S4 Table — (PPTX) [file pone.0280410.s005.pptx]

## Slide 1
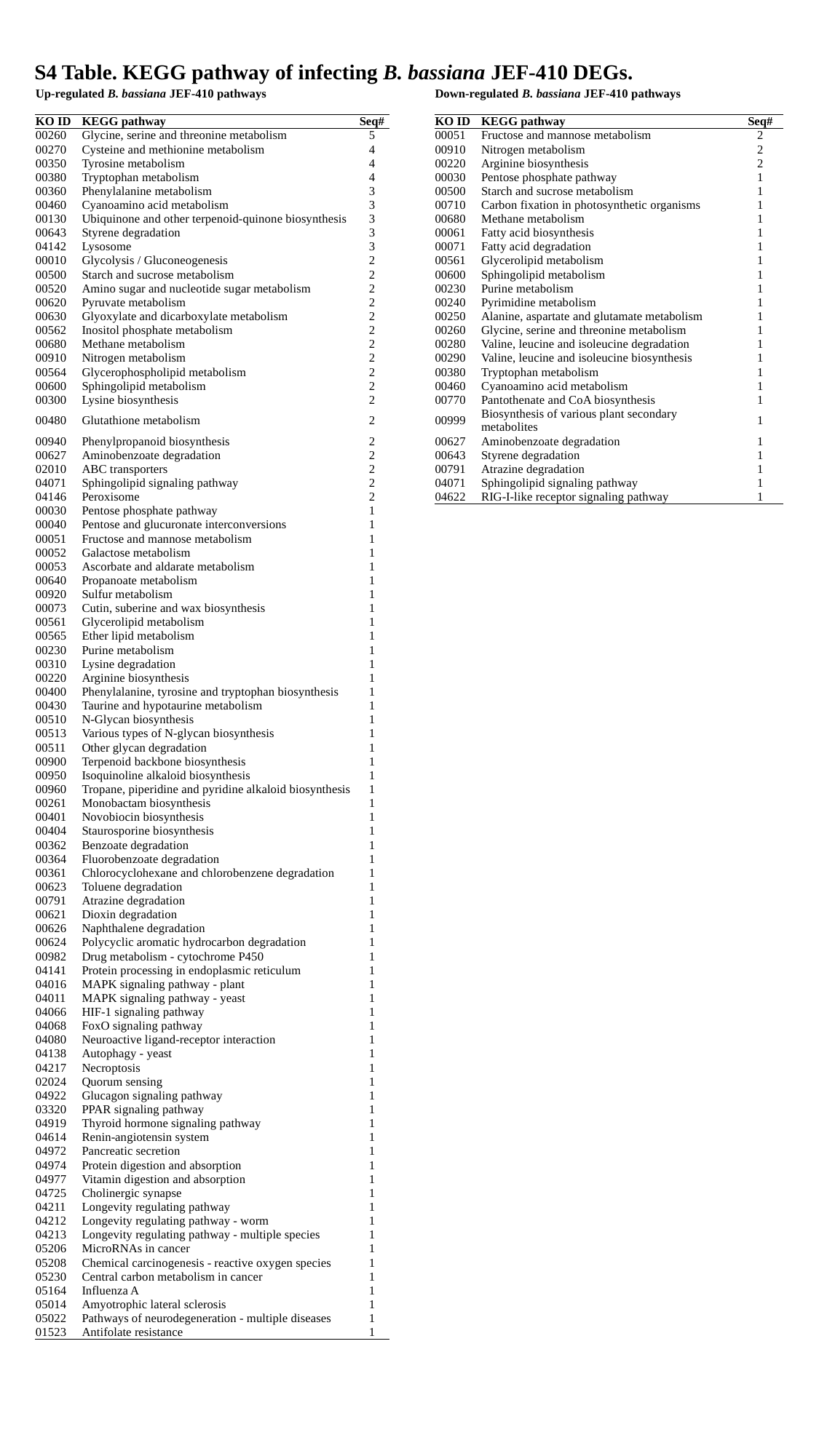

S4 Table. KEGG pathway of infecting B. bassiana JEF-410 DEGs.
| | | | | | | |
| --- | --- | --- | --- | --- | --- | --- |
| Up-regulated B. bassiana JEF-410 pathways | | | | Down-regulated B. bassiana JEF-410 pathways | | |
| | | | | | | |
| KO ID | KEGG pathway | Seq# | | KO ID | KEGG pathway | Seq# |
| 00260 | Glycine, serine and threonine metabolism | 5 | | 00051 | Fructose and mannose metabolism | 2 |
| 00270 | Cysteine and methionine metabolism | 4 | | 00910 | Nitrogen metabolism | 2 |
| 00350 | Tyrosine metabolism | 4 | | 00220 | Arginine biosynthesis | 2 |
| 00380 | Tryptophan metabolism | 4 | | 00030 | Pentose phosphate pathway | 1 |
| 00360 | Phenylalanine metabolism | 3 | | 00500 | Starch and sucrose metabolism | 1 |
| 00460 | Cyanoamino acid metabolism | 3 | | 00710 | Carbon fixation in photosynthetic organisms | 1 |
| 00130 | Ubiquinone and other terpenoid-quinone biosynthesis | 3 | | 00680 | Methane metabolism | 1 |
| 00643 | Styrene degradation | 3 | | 00061 | Fatty acid biosynthesis | 1 |
| 04142 | Lysosome | 3 | | 00071 | Fatty acid degradation | 1 |
| 00010 | Glycolysis / Gluconeogenesis | 2 | | 00561 | Glycerolipid metabolism | 1 |
| 00500 | Starch and sucrose metabolism | 2 | | 00600 | Sphingolipid metabolism | 1 |
| 00520 | Amino sugar and nucleotide sugar metabolism | 2 | | 00230 | Purine metabolism | 1 |
| 00620 | Pyruvate metabolism | 2 | | 00240 | Pyrimidine metabolism | 1 |
| 00630 | Glyoxylate and dicarboxylate metabolism | 2 | | 00250 | Alanine, aspartate and glutamate metabolism | 1 |
| 00562 | Inositol phosphate metabolism | 2 | | 00260 | Glycine, serine and threonine metabolism | 1 |
| 00680 | Methane metabolism | 2 | | 00280 | Valine, leucine and isoleucine degradation | 1 |
| 00910 | Nitrogen metabolism | 2 | | 00290 | Valine, leucine and isoleucine biosynthesis | 1 |
| 00564 | Glycerophospholipid metabolism | 2 | | 00380 | Tryptophan metabolism | 1 |
| 00600 | Sphingolipid metabolism | 2 | | 00460 | Cyanoamino acid metabolism | 1 |
| 00300 | Lysine biosynthesis | 2 | | 00770 | Pantothenate and CoA biosynthesis | 1 |
| 00480 | Glutathione metabolism | 2 | | 00999 | Biosynthesis of various plant secondary metabolites | 1 |
| 00940 | Phenylpropanoid biosynthesis | 2 | | 00627 | Aminobenzoate degradation | 1 |
| 00627 | Aminobenzoate degradation | 2 | | 00643 | Styrene degradation | 1 |
| 02010 | ABC transporters | 2 | | 00791 | Atrazine degradation | 1 |
| 04071 | Sphingolipid signaling pathway | 2 | | 04071 | Sphingolipid signaling pathway | 1 |
| 04146 | Peroxisome | 2 | | 04622 | RIG-I-like receptor signaling pathway | 1 |
| 00030 | Pentose phosphate pathway | 1 | | | | |
| 00040 | Pentose and glucuronate interconversions | 1 | | | | |
| 00051 | Fructose and mannose metabolism | 1 | | | | |
| 00052 | Galactose metabolism | 1 | | | | |
| 00053 | Ascorbate and aldarate metabolism | 1 | | | | |
| 00640 | Propanoate metabolism | 1 | | | | |
| 00920 | Sulfur metabolism | 1 | | | | |
| 00073 | Cutin, suberine and wax biosynthesis | 1 | | | | |
| 00561 | Glycerolipid metabolism | 1 | | | | |
| 00565 | Ether lipid metabolism | 1 | | | | |
| 00230 | Purine metabolism | 1 | | | | |
| 00310 | Lysine degradation | 1 | | | | |
| 00220 | Arginine biosynthesis | 1 | | | | |
| 00400 | Phenylalanine, tyrosine and tryptophan biosynthesis | 1 | | | | |
| 00430 | Taurine and hypotaurine metabolism | 1 | | | | |
| 00510 | N-Glycan biosynthesis | 1 | | | | |
| 00513 | Various types of N-glycan biosynthesis | 1 | | | | |
| 00511 | Other glycan degradation | 1 | | | | |
| 00900 | Terpenoid backbone biosynthesis | 1 | | | | |
| 00950 | Isoquinoline alkaloid biosynthesis | 1 | | | | |
| 00960 | Tropane, piperidine and pyridine alkaloid biosynthesis | 1 | | | | |
| 00261 | Monobactam biosynthesis | 1 | | | | |
| 00401 | Novobiocin biosynthesis | 1 | | | | |
| 00404 | Staurosporine biosynthesis | 1 | | | | |
| 00362 | Benzoate degradation | 1 | | | | |
| 00364 | Fluorobenzoate degradation | 1 | | | | |
| 00361 | Chlorocyclohexane and chlorobenzene degradation | 1 | | | | |
| 00623 | Toluene degradation | 1 | | | | |
| 00791 | Atrazine degradation | 1 | | | | |
| 00621 | Dioxin degradation | 1 | | | | |
| 00626 | Naphthalene degradation | 1 | | | | |
| 00624 | Polycyclic aromatic hydrocarbon degradation | 1 | | | | |
| 00982 | Drug metabolism - cytochrome P450 | 1 | | | | |
| 04141 | Protein processing in endoplasmic reticulum | 1 | | | | |
| 04016 | MAPK signaling pathway - plant | 1 | | | | |
| 04011 | MAPK signaling pathway - yeast | 1 | | | | |
| 04066 | HIF-1 signaling pathway | 1 | | | | |
| 04068 | FoxO signaling pathway | 1 | | | | |
| 04080 | Neuroactive ligand-receptor interaction | 1 | | | | |
| 04138 | Autophagy - yeast | 1 | | | | |
| 04217 | Necroptosis | 1 | | | | |
| 02024 | Quorum sensing | 1 | | | | |
| 04922 | Glucagon signaling pathway | 1 | | | | |
| 03320 | PPAR signaling pathway | 1 | | | | |
| 04919 | Thyroid hormone signaling pathway | 1 | | | | |
| 04614 | Renin-angiotensin system | 1 | | | | |
| 04972 | Pancreatic secretion | 1 | | | | |
| 04974 | Protein digestion and absorption | 1 | | | | |
| 04977 | Vitamin digestion and absorption | 1 | | | | |
| 04725 | Cholinergic synapse | 1 | | | | |
| 04211 | Longevity regulating pathway | 1 | | | | |
| 04212 | Longevity regulating pathway - worm | 1 | | | | |
| 04213 | Longevity regulating pathway - multiple species | 1 | | | | |
| 05206 | MicroRNAs in cancer | 1 | | | | |
| 05208 | Chemical carcinogenesis - reactive oxygen species | 1 | | | | |
| 05230 | Central carbon metabolism in cancer | 1 | | | | |
| 05164 | Influenza A | 1 | | | | |
| 05014 | Amyotrophic lateral sclerosis | 1 | | | | |
| 05022 | Pathways of neurodegeneration - multiple diseases | 1 | | | | |
| 01523 | Antifolate resistance | 1 | | | | |
