# Supplementary material for: Pathogenesis and defense mechanism while Beauveria bassiana JEF-410 infects poultry red mite, Dermanyssus gallinae
Source: PLoS One. 2023 Feb 17;18(2):e0280410. doi: 10.1371/journal.pone.0280410 (PMC9937463; doi:10.1371/journal.pone.0280410)
Supplement: S5 Table — (PPTX) [file pone.0280410.s006.pptx]

## Slide 1
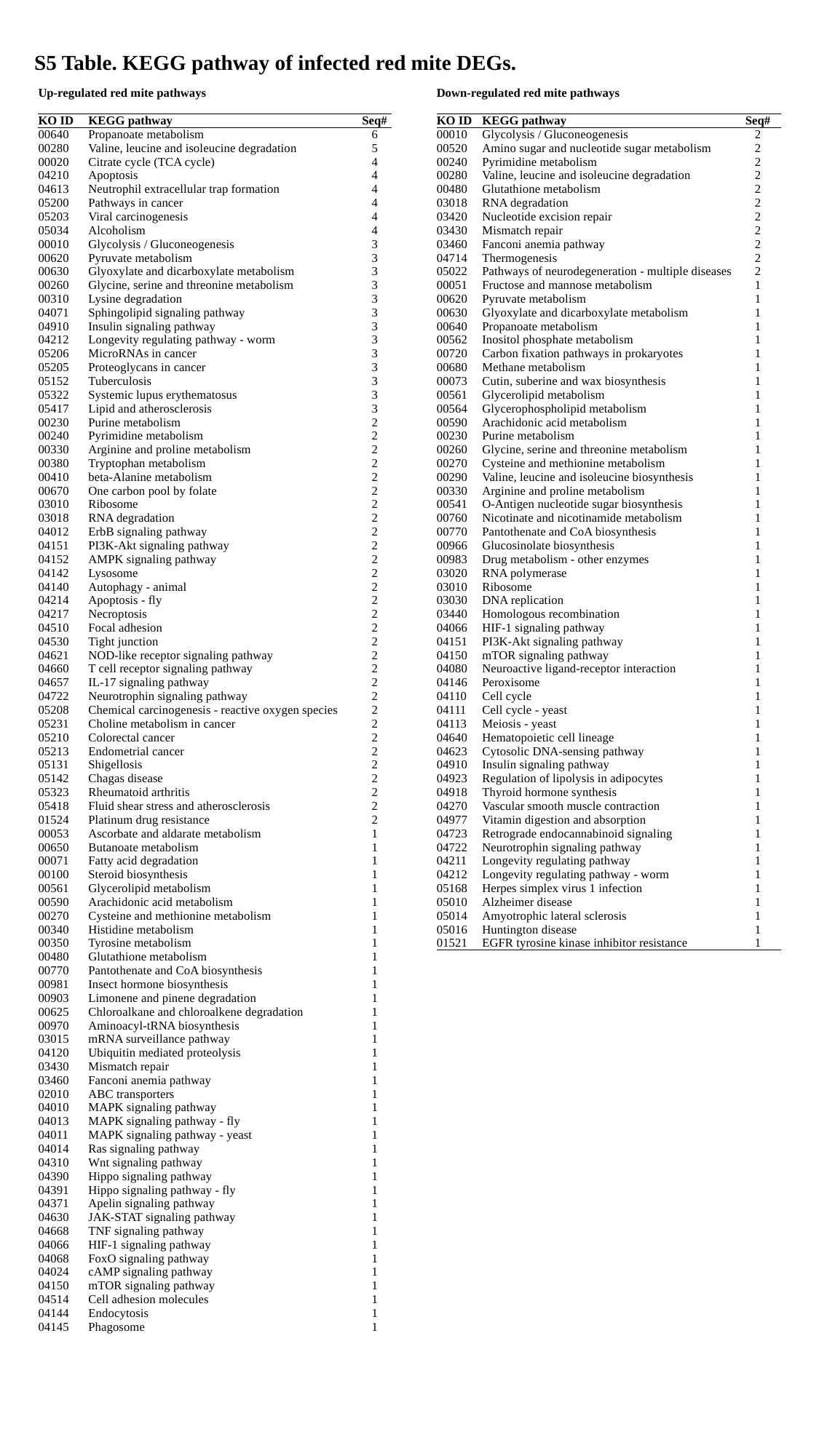

S5 Table. KEGG pathway of infected red mite DEGs.
| | | | | | | |
| --- | --- | --- | --- | --- | --- | --- |
| Up-regulated red mite pathways | | | | Down-regulated red mite pathways | | |
| | | | | | | |
| KO ID | KEGG pathway | Seq# | | KO ID | KEGG pathway | Seq# |
| 00640 | Propanoate metabolism | 6 | | 00010 | Glycolysis / Gluconeogenesis | 2 |
| 00280 | Valine, leucine and isoleucine degradation | 5 | | 00520 | Amino sugar and nucleotide sugar metabolism | 2 |
| 00020 | Citrate cycle (TCA cycle) | 4 | | 00240 | Pyrimidine metabolism | 2 |
| 04210 | Apoptosis | 4 | | 00280 | Valine, leucine and isoleucine degradation | 2 |
| 04613 | Neutrophil extracellular trap formation | 4 | | 00480 | Glutathione metabolism | 2 |
| 05200 | Pathways in cancer | 4 | | 03018 | RNA degradation | 2 |
| 05203 | Viral carcinogenesis | 4 | | 03420 | Nucleotide excision repair | 2 |
| 05034 | Alcoholism | 4 | | 03430 | Mismatch repair | 2 |
| 00010 | Glycolysis / Gluconeogenesis | 3 | | 03460 | Fanconi anemia pathway | 2 |
| 00620 | Pyruvate metabolism | 3 | | 04714 | Thermogenesis | 2 |
| 00630 | Glyoxylate and dicarboxylate metabolism | 3 | | 05022 | Pathways of neurodegeneration - multiple diseases | 2 |
| 00260 | Glycine, serine and threonine metabolism | 3 | | 00051 | Fructose and mannose metabolism | 1 |
| 00310 | Lysine degradation | 3 | | 00620 | Pyruvate metabolism | 1 |
| 04071 | Sphingolipid signaling pathway | 3 | | 00630 | Glyoxylate and dicarboxylate metabolism | 1 |
| 04910 | Insulin signaling pathway | 3 | | 00640 | Propanoate metabolism | 1 |
| 04212 | Longevity regulating pathway - worm | 3 | | 00562 | Inositol phosphate metabolism | 1 |
| 05206 | MicroRNAs in cancer | 3 | | 00720 | Carbon fixation pathways in prokaryotes | 1 |
| 05205 | Proteoglycans in cancer | 3 | | 00680 | Methane metabolism | 1 |
| 05152 | Tuberculosis | 3 | | 00073 | Cutin, suberine and wax biosynthesis | 1 |
| 05322 | Systemic lupus erythematosus | 3 | | 00561 | Glycerolipid metabolism | 1 |
| 05417 | Lipid and atherosclerosis | 3 | | 00564 | Glycerophospholipid metabolism | 1 |
| 00230 | Purine metabolism | 2 | | 00590 | Arachidonic acid metabolism | 1 |
| 00240 | Pyrimidine metabolism | 2 | | 00230 | Purine metabolism | 1 |
| 00330 | Arginine and proline metabolism | 2 | | 00260 | Glycine, serine and threonine metabolism | 1 |
| 00380 | Tryptophan metabolism | 2 | | 00270 | Cysteine and methionine metabolism | 1 |
| 00410 | beta-Alanine metabolism | 2 | | 00290 | Valine, leucine and isoleucine biosynthesis | 1 |
| 00670 | One carbon pool by folate | 2 | | 00330 | Arginine and proline metabolism | 1 |
| 03010 | Ribosome | 2 | | 00541 | O-Antigen nucleotide sugar biosynthesis | 1 |
| 03018 | RNA degradation | 2 | | 00760 | Nicotinate and nicotinamide metabolism | 1 |
| 04012 | ErbB signaling pathway | 2 | | 00770 | Pantothenate and CoA biosynthesis | 1 |
| 04151 | PI3K-Akt signaling pathway | 2 | | 00966 | Glucosinolate biosynthesis | 1 |
| 04152 | AMPK signaling pathway | 2 | | 00983 | Drug metabolism - other enzymes | 1 |
| 04142 | Lysosome | 2 | | 03020 | RNA polymerase | 1 |
| 04140 | Autophagy - animal | 2 | | 03010 | Ribosome | 1 |
| 04214 | Apoptosis - fly | 2 | | 03030 | DNA replication | 1 |
| 04217 | Necroptosis | 2 | | 03440 | Homologous recombination | 1 |
| 04510 | Focal adhesion | 2 | | 04066 | HIF-1 signaling pathway | 1 |
| 04530 | Tight junction | 2 | | 04151 | PI3K-Akt signaling pathway | 1 |
| 04621 | NOD-like receptor signaling pathway | 2 | | 04150 | mTOR signaling pathway | 1 |
| 04660 | T cell receptor signaling pathway | 2 | | 04080 | Neuroactive ligand-receptor interaction | 1 |
| 04657 | IL-17 signaling pathway | 2 | | 04146 | Peroxisome | 1 |
| 04722 | Neurotrophin signaling pathway | 2 | | 04110 | Cell cycle | 1 |
| 05208 | Chemical carcinogenesis - reactive oxygen species | 2 | | 04111 | Cell cycle - yeast | 1 |
| 05231 | Choline metabolism in cancer | 2 | | 04113 | Meiosis - yeast | 1 |
| 05210 | Colorectal cancer | 2 | | 04640 | Hematopoietic cell lineage | 1 |
| 05213 | Endometrial cancer | 2 | | 04623 | Cytosolic DNA-sensing pathway | 1 |
| 05131 | Shigellosis | 2 | | 04910 | Insulin signaling pathway | 1 |
| 05142 | Chagas disease | 2 | | 04923 | Regulation of lipolysis in adipocytes | 1 |
| 05323 | Rheumatoid arthritis | 2 | | 04918 | Thyroid hormone synthesis | 1 |
| 05418 | Fluid shear stress and atherosclerosis | 2 | | 04270 | Vascular smooth muscle contraction | 1 |
| 01524 | Platinum drug resistance | 2 | | 04977 | Vitamin digestion and absorption | 1 |
| 00053 | Ascorbate and aldarate metabolism | 1 | | 04723 | Retrograde endocannabinoid signaling | 1 |
| 00650 | Butanoate metabolism | 1 | | 04722 | Neurotrophin signaling pathway | 1 |
| 00071 | Fatty acid degradation | 1 | | 04211 | Longevity regulating pathway | 1 |
| 00100 | Steroid biosynthesis | 1 | | 04212 | Longevity regulating pathway - worm | 1 |
| 00561 | Glycerolipid metabolism | 1 | | 05168 | Herpes simplex virus 1 infection | 1 |
| 00590 | Arachidonic acid metabolism | 1 | | 05010 | Alzheimer disease | 1 |
| 00270 | Cysteine and methionine metabolism | 1 | | 05014 | Amyotrophic lateral sclerosis | 1 |
| 00340 | Histidine metabolism | 1 | | 05016 | Huntington disease | 1 |
| 00350 | Tyrosine metabolism | 1 | | 01521 | EGFR tyrosine kinase inhibitor resistance | 1 |
| 00480 | Glutathione metabolism | 1 | | | | |
| 00770 | Pantothenate and CoA biosynthesis | 1 | | | | |
| 00981 | Insect hormone biosynthesis | 1 | | | | |
| 00903 | Limonene and pinene degradation | 1 | | | | |
| 00625 | Chloroalkane and chloroalkene degradation | 1 | | | | |
| 00970 | Aminoacyl-tRNA biosynthesis | 1 | | | | |
| 03015 | mRNA surveillance pathway | 1 | | | | |
| 04120 | Ubiquitin mediated proteolysis | 1 | | | | |
| 03430 | Mismatch repair | 1 | | | | |
| 03460 | Fanconi anemia pathway | 1 | | | | |
| 02010 | ABC transporters | 1 | | | | |
| 04010 | MAPK signaling pathway | 1 | | | | |
| 04013 | MAPK signaling pathway - fly | 1 | | | | |
| 04011 | MAPK signaling pathway - yeast | 1 | | | | |
| 04014 | Ras signaling pathway | 1 | | | | |
| 04310 | Wnt signaling pathway | 1 | | | | |
| 04390 | Hippo signaling pathway | 1 | | | | |
| 04391 | Hippo signaling pathway - fly | 1 | | | | |
| 04371 | Apelin signaling pathway | 1 | | | | |
| 04630 | JAK-STAT signaling pathway | 1 | | | | |
| 04668 | TNF signaling pathway | 1 | | | | |
| 04066 | HIF-1 signaling pathway | 1 | | | | |
| 04068 | FoxO signaling pathway | 1 | | | | |
| 04024 | cAMP signaling pathway | 1 | | | | |
| 04150 | mTOR signaling pathway | 1 | | | | |
| 04514 | Cell adhesion molecules | 1 | | | | |
| 04144 | Endocytosis | 1 | | | | |
| 04145 | Phagosome | 1 | | | | |

## Slide 2
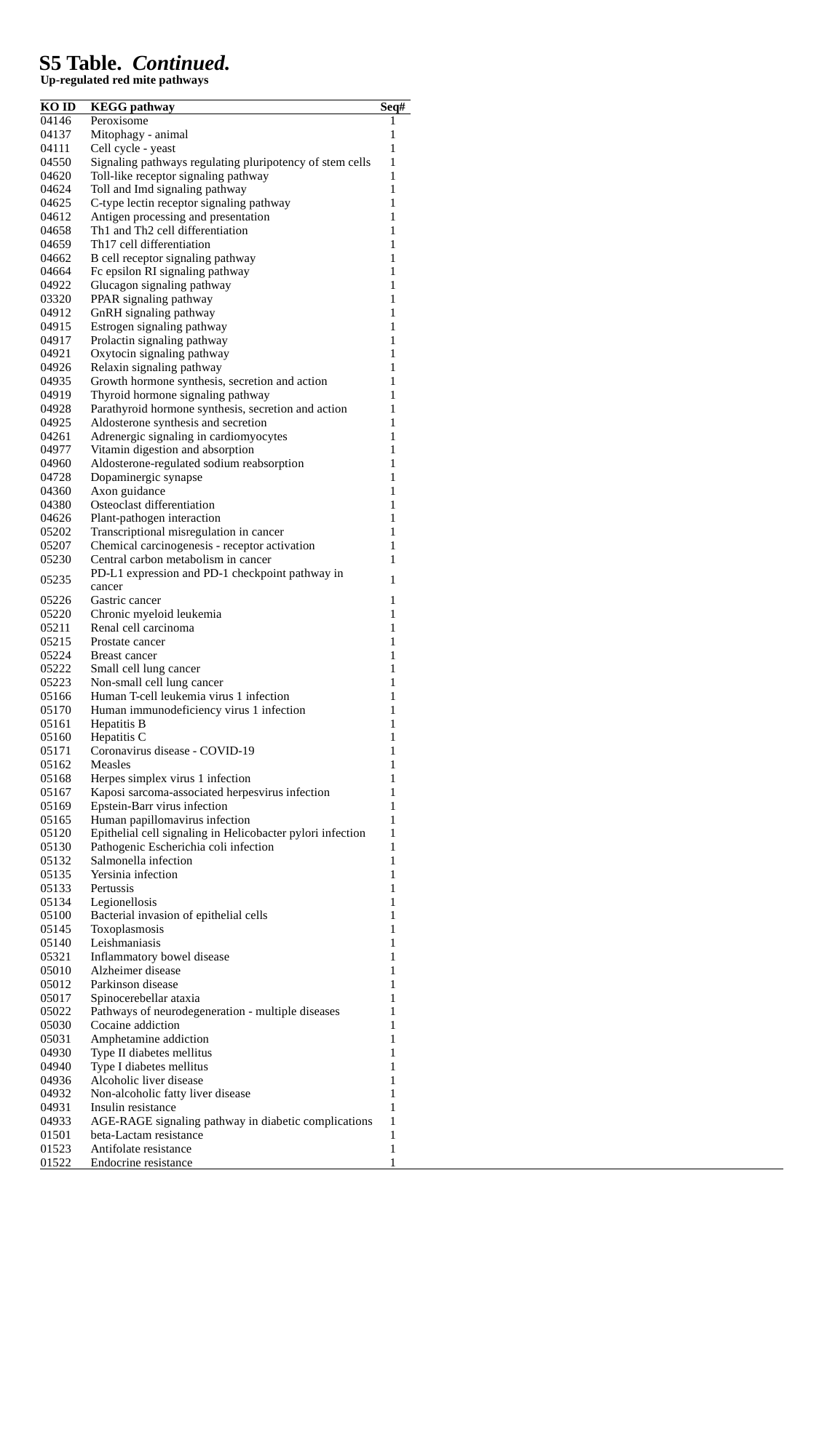

S5 Table. Continued.
| | | | | | | |
| --- | --- | --- | --- | --- | --- | --- |
| Up-regulated red mite pathways | | | | | | |
| | | | | | | |
| KO ID | KEGG pathway | Seq# | | | | |
| 04146 | Peroxisome | 1 | | | | |
| 04137 | Mitophagy - animal | 1 | | | | |
| 04111 | Cell cycle - yeast | 1 | | | | |
| 04550 | Signaling pathways regulating pluripotency of stem cells | 1 | | | | |
| 04620 | Toll-like receptor signaling pathway | 1 | | | | |
| 04624 | Toll and Imd signaling pathway | 1 | | | | |
| 04625 | C-type lectin receptor signaling pathway | 1 | | | | |
| 04612 | Antigen processing and presentation | 1 | | | | |
| 04658 | Th1 and Th2 cell differentiation | 1 | | | | |
| 04659 | Th17 cell differentiation | 1 | | | | |
| 04662 | B cell receptor signaling pathway | 1 | | | | |
| 04664 | Fc epsilon RI signaling pathway | 1 | | | | |
| 04922 | Glucagon signaling pathway | 1 | | | | |
| 03320 | PPAR signaling pathway | 1 | | | | |
| 04912 | GnRH signaling pathway | 1 | | | | |
| 04915 | Estrogen signaling pathway | 1 | | | | |
| 04917 | Prolactin signaling pathway | 1 | | | | |
| 04921 | Oxytocin signaling pathway | 1 | | | | |
| 04926 | Relaxin signaling pathway | 1 | | | | |
| 04935 | Growth hormone synthesis, secretion and action | 1 | | | | |
| 04919 | Thyroid hormone signaling pathway | 1 | | | | |
| 04928 | Parathyroid hormone synthesis, secretion and action | 1 | | | | |
| 04925 | Aldosterone synthesis and secretion | 1 | | | | |
| 04261 | Adrenergic signaling in cardiomyocytes | 1 | | | | |
| 04977 | Vitamin digestion and absorption | 1 | | | | |
| 04960 | Aldosterone-regulated sodium reabsorption | 1 | | | | |
| 04728 | Dopaminergic synapse | 1 | | | | |
| 04360 | Axon guidance | 1 | | | | |
| 04380 | Osteoclast differentiation | 1 | | | | |
| 04626 | Plant-pathogen interaction | 1 | | | | |
| 05202 | Transcriptional misregulation in cancer | 1 | | | | |
| 05207 | Chemical carcinogenesis - receptor activation | 1 | | | | |
| 05230 | Central carbon metabolism in cancer | 1 | | | | |
| 05235 | PD-L1 expression and PD-1 checkpoint pathway in cancer | 1 | | | | |
| 05226 | Gastric cancer | 1 | | | | |
| 05220 | Chronic myeloid leukemia | 1 | | | | |
| 05211 | Renal cell carcinoma | 1 | | | | |
| 05215 | Prostate cancer | 1 | | | | |
| 05224 | Breast cancer | 1 | | | | |
| 05222 | Small cell lung cancer | 1 | | | | |
| 05223 | Non-small cell lung cancer | 1 | | | | |
| 05166 | Human T-cell leukemia virus 1 infection | 1 | | | | |
| 05170 | Human immunodeficiency virus 1 infection | 1 | | | | |
| 05161 | Hepatitis B | 1 | | | | |
| 05160 | Hepatitis C | 1 | | | | |
| 05171 | Coronavirus disease - COVID-19 | 1 | | | | |
| 05162 | Measles | 1 | | | | |
| 05168 | Herpes simplex virus 1 infection | 1 | | | | |
| 05167 | Kaposi sarcoma-associated herpesvirus infection | 1 | | | | |
| 05169 | Epstein-Barr virus infection | 1 | | | | |
| 05165 | Human papillomavirus infection | 1 | | | | |
| 05120 | Epithelial cell signaling in Helicobacter pylori infection | 1 | | | | |
| 05130 | Pathogenic Escherichia coli infection | 1 | | | | |
| 05132 | Salmonella infection | 1 | | | | |
| 05135 | Yersinia infection | 1 | | | | |
| 05133 | Pertussis | 1 | | | | |
| 05134 | Legionellosis | 1 | | | | |
| 05100 | Bacterial invasion of epithelial cells | 1 | | | | |
| 05145 | Toxoplasmosis | 1 | | | | |
| 05140 | Leishmaniasis | 1 | | | | |
| 05321 | Inflammatory bowel disease | 1 | | | | |
| 05010 | Alzheimer disease | 1 | | | | |
| 05012 | Parkinson disease | 1 | | | | |
| 05017 | Spinocerebellar ataxia | 1 | | | | |
| 05022 | Pathways of neurodegeneration - multiple diseases | 1 | | | | |
| 05030 | Cocaine addiction | 1 | | | | |
| 05031 | Amphetamine addiction | 1 | | | | |
| 04930 | Type II diabetes mellitus | 1 | | | | |
| 04940 | Type I diabetes mellitus | 1 | | | | |
| 04936 | Alcoholic liver disease | 1 | | | | |
| 04932 | Non-alcoholic fatty liver disease | 1 | | | | |
| 04931 | Insulin resistance | 1 | | | | |
| 04933 | AGE-RAGE signaling pathway in diabetic complications | 1 | | | | |
| 01501 | beta-Lactam resistance | 1 | | | | |
| 01523 | Antifolate resistance | 1 | | | | |
| 01522 | Endocrine resistance | 1 | | | | |
