# Supplementary material for: Pathogenesis and defense mechanism while Beauveria bassiana JEF-410 infects poultry red mite, Dermanyssus gallinae
Source: PLoS One. 2023 Feb 17;18(2):e0280410. doi: 10.1371/journal.pone.0280410 (PMC9937463; doi:10.1371/journal.pone.0280410)
Supplement: Attachment — Submitted filename: 06. Response to Comments_final.docx [file pone.0280410.s007.docx]

Reviewer's Responses to Questions

Review Comments to the Author

Please use the space provided to explain your answers to the questions above. You may also include additional comments for the author, including concerns about dual publication, research ethics, or publication ethics. (Please upload your review as an attachment if it exceeds 20,000 characters)

→ I really appreciate your comments and recommendation. Per your comments I revised this manuscript very carefully.

Reviewer #1: The work is novel and explores the interactions that exist between the entomopathogenic fungus and its host red mites. By elucidating these interactions, it is possible to optimize the insecticidal action of the fungus, either by inducing or overexpressing insecticidal agents or by reducing the insect's defense tools.

However, I have some observations:

→ Thank you! As commented, I described these interactions much clear in the revised manuscript.

1. The introduction adequately contextualizes the red mite pest. However, it does not clearly indicate which are the advances described in the literature about the interactions between entomopathogenic fungus and insect.

1.1. In lines 68-70, a work is cited that studies the transcriptome of two-spotted spider mite, however, this study is about the expression of genes in diapause. Therefore, it does not seem pertinent to cite it without informing that it is in this specific state. Besides, red mites do not go through this stage.

→ As commented, the description of the two-spotted spider mite were deleted and changed with more suitable information.

1.2 The pathogenicity mechanisms of the fungus and the defense mechanisms of the insect should be specified in order to provide the reader with guidelines for the work. What factors does the fungus secrete? What are the defense mechanisms of the insect?

→ As commented, the description was modified and updated with a resistance mechanism of tick against fungal pathogenesis during attack.

2. The methodology is very well explained and the statistical analyses correspond to the type of sample.

However:

2.1. In lines: 97, 98, 120, 127, 203, 203, 204, 205, 209, 210 and 211, separate degrees from the unit celsius (e.g. 20° C).

→ As commented, I changed all symbols to °C.

2.2. On line 166 correct URL: https://bioconductor.org/packages/devel/bioc/html/edgeR.html.

→ As commented, I changed the URL address.

3. The results are well written, just consider these remarks:

3.1 In line 274: add period after 53.0%.

→ As commented, I corrected it with period marking.

3.2 In Figure 1C, 5 and 6: add meaning of the abbreviation "1R", "2R", "3R".

→ As commented, I added the meaning of “R” on figure 1: The meaning of the ‘R’ attached to each number is the repetition of the sample.

3.3 In Figure 3: complete the sentences, because it is necessary to specify what each of the evaluated gene groups refers to. The figure should provide all the information by itself.

→ As commented, I updated the figure legends in figure 3 and 4 to provide the complete information in the sentence itself.

4. With respect to the results, I consider that some of the conclusions are somewhat hasty. In addition, the information that is taken for granted was not referenced.

4.1. Line 330: The title does not cover what is addressed in the body of the text. If you intend to establish that the mechanism of tryptophan is in the early stages of infection.... What is the range or stages that this encompasses? It should be contextualized as to what "early stage" refers to. Personally, I would not state it as part of the title of the result, since one could not be sure that it is in the early stage. That is mostly a conclusion formulated from the literature, but not from the experiment itself.

→ In our previous experiment (published data at <https://doi.org/10.1007/s10526-021-10110-w> (Park et al., BioControl, 2021)), LT_25_ of *B. bassiana* JEF 410 at 1x10^7^ conidia/ml was about 2.5 days after treatment. Based on this data, we determined it as an early stage of infection.

4.2. In line 335-335, I would eliminate this sentence, since it is arbitrary. Neither in the introduction or results have they pointed out why tryptophan metabolism is relevant. I consider that eliminating it and starting with the following paragraph is the most appropriate.

→ As commented, I deleted it and started with the suggestion.

4.3. In lines 340-347, add a reference that evidences what has been pointed out.

→ As commented, I added a reference to support it.

4.4 In line 380: I would not use "Suggests", since the mechanism of infection is highly evidenced, especially of Beauveria bassiana. So it is a fact that it invades the hemolymph, by penetration of the epicuticle. Use the word "confirms" instead.

→ As commented, I revised it.

4.5 On line 382: Change sentence to "The fungi that invade the cuticle attack the host...".

→ As commented, I revised the sentence.

4.6 On line 452: The conclusion seems unfounded, since one could not speak of promoting. Only that in conjunction with the increase of genes coding for secondary metabolites, it could be established that secondary metabolites are relevant for the infection of the fungus in red mites. The word promote implies temporal order, and neither result accounts for that.

→ As you commented, we revised it as follows: It is likely that the increase in gene expression of secondary metabolites such as beauvericin, beauveriolides, and bassianolide of *B. bassiana* JEF-410 is probably involved in the infection of red mites.

Reviewer #2: In this paper，the pathogenesis of the acaricidal fungus Beauveria bassiana JEF-410 and the

defense mechanisms of red mites were studied by transcriptome analysis. Although the results presented here are intriguing, numerous grave issues still exist.

Major concerns:

1. The strain name needs to be consistently shortened throughout the text. JEF-410 is referred to in lines 25, 28, and 39, but B. bassiana JEF-410 is found in line 29.

→ As commented, I made the strain name shortened uniformly throughout the revised manuscript.

2. Lines 68-70, reference 14 should be added at the end of the sentence “suppression of up-regulated genes suggests enhanced insecticidal activity.” However, I couldn’t find the relative information as this paper described in reference 14, please check again.

→ As commented, I updated the reference to make it strongly support our data and located at the end of the sentence.

3. The bar chart's color in figures 2, 3, and 4 is not appealing.

→ As commented, I made the figures in black and white mode.

4. Lines 240-249, results regarding the heat map of B. bassiana JEF-410 and red mite (Fig 1C) were inconclusive. Additionally, certain information should be included in the figure1 illustration, such as descriptions of 1R, 2R, and 3R. Why is the B. bassiana JEF-410 control group experiment lacking in 3R data?

→ Your comment could be right, but in this analysis of heatmap I tried to investigate the variation of gene expression among the repetitions in each treatment. And the heatmaps showed a quite similar patterns of expression in each treatment.

→ I sequenced the 3^rd^ replicate (3R) of the non-infection JEF-410 but the gene expression pattern was significantly different from the other two replicates, so unfortunately 3R was exclusive from this analysis.

5. Line 269, “In the GO analysis of red mite,” should be red mites.

→ As commented, I corrected it as red mites.

6. Line 340, the author claimed that overexpressed enzymes had no significant effect on pathogenicity, although analysis revealed that synthesizing acetyl-CoA and is involved in various cellular processes required for infection (lines 358-359). Some of the conclusions are in disagreement. Please elaborate.

→ Your comment is right. To clarify it, I deleted the sentence (overexpressed enzymes had no significant effect on pathogenicity).

7. Relevant references should be added to lines 340–344.

→ As commented, I added relevant references.

8. More detailed information has to be provided because the subject on this page is somewhat broad. Instead of simply discussing their metabolic pathways in general, what are acetyl-CoA and certain energy metabolism-related genes in B. bassiana JEF-410 infecting red mites, and how frequently are they up-regulated or down-regulated?

→ Thank you for your good comments! I provided major genes from metabolism for more deep understanding. As presented in GO enrichment analysis of Figure 4, the most up-regulated pathways in the infecting JEF-410 were metabolic pathway (KEGG: 01100), followed by tryptophan metabolism (KEGG:00380). Significantly up-regulated genes in the tryptophan metabolism were YBL098W, YIL164C, YGR088W, and YGL202W. Each gene performs the functions of kynurenine 3-monooxygenase, nitrilase, and cytosolic catalase T. The tryptophan metabolism performs energy production by synthesizing kynurenine followed by NAD synthesis or by synthesizing Acetyl-CoA. It was confirmed that the related pathway was up-regulated by validation here in this work.

→ In this work, I extracted a total of RNA in 2.5 days after fungal treatment, so I could just describe the up- and down-regulation in the determined time point. However, as the pathogenesis is proceeding, the pathogenesis-related genes would be more highly up-regulated and finally back to normal expression when the fungal infection and propagation is over.
